# Brain functional connectivity changes in amyotrophic lateral sclerosis with apathy and depression

**DOI:** 10.1007/s00415-025-13247-1

**Published:** 2025-07-14

**Authors:** Veronica Castelnovo, Elisa Canu, Silvia Basaia, Edoardo Gioele Spinelli, Fabiola Freri, Paride Schito, Tommaso Russo, Yuri Falzone, Federico Verde, Silvia Torre, Barbara Poletti, Lucio Tremolizzo, Ildebrando Appollonio, Nicola Ticozzi, Vincenzo Silani, Massimo Filippi, Federica Agosta

**Affiliations:** 1 Neuroimaging Research Unit, Division of Neuroscience, IRCCS San Raffaele Scientific Institute, Milan, Italy; 2 Neurology Unit, IRCCS San Raffaele Scientific Institute, Milan, Italy; 3 Vita-Salute San Raffaele University, Milan, Italy; 4 Department of Neurology and Laboratory of Neuroscience, IRCCS Istituto Auxologico Italiano, Milan, Italy; 5 Department of Pathophysiology and Transplantation, Dino Ferrari” Center, Università Degli Studi Di Milano, Milan, Italy; 6 Department of Oncology and Hemato-Oncology, Università Degli Studi Di Milano, Milan, Italy; 7 Neurology Unit, “San Gerardo” Hospital and University of Milano-Bicocca, Monza, Italy; 8 Neurorehabilitation Unit, IRCCS San Raffaele Scientific Institute, Milan, Italy; 9 Neurophysiology Service, IRCCS San Raffaele Scientific Institute, Milan, Italy

**Keywords:** Amyotrophic lateral sclerosis, Apathy, Depression, Graph analysis, Functional connectivity, Resting-state fMRI

## Abstract

**Background:**

Apathy and depression are the most prevalent neuropsychiatric symptoms in amyotrophic lateral sclerosis (ALS). Although insufficiently investigated, their distinction holds important clinical relevance for accurate diagnosis of ALS with behavioural impairment and for patients’ prognosis and management. In the present study, we aimed to assess both apathy and depressive symptoms in patients with ALS and whether they have similar or different functional neural correlates.

**Methods:**

Using graph analysis and connectomics, global and lobar nodal properties and regional functional brain connectivity were assessed in ALS patients without apathy/depression (ALSn, *n* = 42), with apathy without depression (ALSa, *n* = 14), with depressive symptoms without apathy (ALSd, *n* = 20), and with apathy and depressive symptoms (ALSad, *n* = 6), and 46 healthy controls. Correlations between brain functional properties, apathy and depressive symptoms were performed in all patients.

**Results:**

Depressive symptoms were related with reduced path length within bilateral basal ganglia (BG) network, and apathy was related with increased path length, decreased nodal strength and local efficiency within left BG network. ALSa patients showed altered functional nodal properties within BG network compared to ALSn and ALSd. Compared to healthy controls and all non-apathetic patients (ALSn and ALSd), all apathetic patients (ALSa and ALSad) exhibited altered functional nodal properties within parietal, occipital and frontal networks. Non-apathetic patients, compared to apathetic patients, showed relatively preserved functional nodal properties in the BG network.

**Conclusions:**

Our findings indicate differences in brain functional neural organization associated with apathy and depression, underscoring the importance of distinguishing these symptoms in ALS and highlighting the need for targeted interventions.

**Supplementary Information:**

The online version contains supplementary material available at 10.1007/s00415-025-13247-1.

## Introduction

Amyotrophic lateral sclerosis (ALS) is a progressive and fatal neurodegenerative disease which affects the motor and extra-motor systems. Extra-motor manifestations occur in about 50% of patients with ALS and include cognitive and/or behavioural changes, depression and anxiety [1]. Cognitive impairment primarily affects executive functions, especially verbal fluency [2]. The most prevalent neuropsychiatric symptoms in ALS patients are apathy (observed in about 30% of patients) [3] and depression (which occurs in 15–25% of cases) [4], followed by loss of sympathy, disinhibition, perseverative and stereotyped behaviors and hyperorality [2].

The presence of apathy has such a relevance in ALS patients that the current extra-motor criteria [5] consider it as a “core symptom” which, alone, leads to a diagnosis of ALS with behavioural impairment (ALSbi). Apathy is a key prognostic factor in ALS [6] and in other syndromes associated with frontotemporal dementia (FTD) [7] and is associated with worse progression [7], disability and functional independence [8]. The presence of apathy in ALS has been associated with reduced brain structural integrity of frontal and parietal regions, particularly the right anterior cingulate and angular gyri [9–11].

In ALS, apathy may present as an isolated behavioural symptom or coexist with depression [4, 12]. Depression, however, can also occur independently in ALS and is often misinterpreted as apathy due to the similarities in their clinical presentations [13]. Depressive symptoms in ALS are partly attributable to the emotional impact of the diagnosis itself. In fact, depression is more likely to develop after ALS onset, particularly within the first year following the appearance of motor symptoms [14]. Depression can introduce numerous health complications, including reduced appetite, impaired sleep, increased social isolation, diminished self-care, and poor adherence to treatment plans. When coupled with ALS, depression can further exacerbate disease progression and negatively affect overall health outcomes [4]. In comparison to apathy, there is limited information about the mechanisms underlying depression in patients with ALS [4]. A longitudinal study found a negative correlation between variations in depressive symptoms over time and changes in cortical thickness in the right orbitofrontal cortex and middle frontal areas in patients with ALS [1]. A recent study using functional connectivity analysis found that ALS patients with depression, compared to controls and to ALS patients without depression, spend more time in a segregated state and less in an integrated state of dynamic network connectivity, though apathy co-presence was not assessed [15].

The clear distinction among apathy and depression, as well as their overlap, has not been thoroughly investigated in ALS. Some studies did not use psychodiagnostic tools to assess depression [16], and others did not differentiate the two symptoms or did not assess apathy while controlling for depression as a covariate in their analyses [9, 11]. Addressing the distinction between apathy and depression holds important clinical relevance for accurate ALSbi diagnosis, patients’ prognosis, behavioural symptom management and for providing family support [5]. In fact, understanding the neural dissociation between these two symptoms in ALS could be key in developing tailored therapeutic interventions targeting specific neural alterations linked to each symptom.

In the present study, we aimed to investigate both apathy and depressive symptoms in patients with ALS and whether they have similar or different functional neural correlates using graph analysis and connectomics.

## Materials and methods

### Participants

From a sample of 368 patients with motor neuron disease, we selected 82 patients who met the clinical criteria for probable or definite ALS [17]. Inclusion was limited to patients who had undergone all the following: a detailed clinical evaluation, a comprehensive neuropsychological assessment (including mood and behavior investigation), and both structural and resting-state functional magnetic resonance imaging (MRI) scans. The included patients were those consecutively evaluated at two ALS Centers (IRCCS Ospedale San Raffaele and IRCCS Istituto Auxologico Italiano) in Milan, Italy, between 2007 and 2022. Forty-six healthy controls were also recruited among non-consanguineous relatives and by word of mouth. Inclusion criteria for healthy controls were the following: Mini-Mental State Examination (MMSE) > 27 [18]; absence of mood disturbances as demonstrated by a score < 9 at the Beck Depression Inventory (BDI) [19] or by an interview with an expert neuropsychologist; no family history for neurodegenerative diseases. Exclusion criteria for all participants were the following: significant medical illnesses or substance abuse which could interfere with cognitive functioning and mood; any (other) major systemic, psychiatric, or neurological illnesses; and (other) causes of focal or diffuse brain damage, including cerebrovascular disease at conventional MRI scans.

Information on handedness was available for 53 ALS patients and 36 healthy controls. All participants were right-handed, except for three ALS patients, two of whom were left-handed and one ambidextrous.

### Clinical assessment

Disease severity was assessed using the revised ALS Functional Rating Scale (ALSFRS-R) [20]. Disease duration was calculated from symptom onset to neuropsychological assessment in months. The disease progression rate was calculated with the following formula: (48–ALSFRS-R score)/disease duration [20].

### Genetic analysis

Genetic analysis was performed in 94% (*N* = 77) of ALS patients. The presence of GGGGCC hexanucleotide expansion in the first intron of *C9ORF72* was assessed using a repeat-primed polymerase chain reaction (PCR) assay [21]. A cut-off of ≥ 30 repeats combined with a typical saw-tooth pattern was considered pathological. In addition, the coding sequences and intron/exon boundaries of *GRN, MAPT, TARDBP* and *SOD1* genes were amplified by PCR using optimized protocols, looking for known pathogenic mutations [22].

### Neuropsychological evaluation

The following cognitive functions were evaluated: global cognitive functioning with the MMSE [18]; long- and short-term verbal memory with the Rey Auditory Verbal Learning Test [23] and the digit span forward [24], respectively; executive functions with the digit span backward [25], the Cognitive Estimation Task [26], the Wisconsin Card Sorting Test [27] or the Modified Card Sorting Test [28]; fluency with the phonemic and semantic fluency tests [29], and the relative fluency indices [30, 31]; language with the naming sub-tests of the Italian battery for the assessment of aphasic disorders (BADA) [32]; mood with the Hamilton Depression Rating Scale (HDRS) [33] and/or the BDI [19]; and the presence of behavioural disturbances with the Amyotrophic Lateral Sclerosis-Frontotemporal Dementia-Questionnaire (ALSFTD-Q) [34] and the Frontal Behavioural Inventory (FBI) [35] administered to patients’ caregivers. As this was a retrospective dataset, 38 out of 42 healthy controls underwent the entire assessment except for the Stroop interference test, the Cognitive Estimation Task, the BADA, and the HDRS. In addition, a subgroup of 28 ALS patients (who were recruited from 2017 onward, shortly after the Italian normative data became available) also underwent the Edinburgh Cognitive and Behavioural ALS Screen (ECAS) [36]. Neuropsychological phenotypes—i.e., ALS cognitively and behaviorally normal (ALScbn), cognitively impaired (ALSci), ALSbi, cognitive and behaviorally impaired (ALScbi) or ALSFTD—were retrieved according to Strong et al.’s [5] criteria.

Patients were considered apathetic if their caregiver provided scores > 1 in the items assessing apathy of FBI (item 1 and 2) and/or ALSFTD-Q (item 1 and 7). In order to have a common measure for apathy to be used for the correlation analysis, a composite measure of apathy was constructed. Z-scores were calculated for items 1 and 2 of the FBI and items 1 and 7 of the ALSFTD-Q. The composite measure “apathy” was obtained from the average of these z-scores. A score greater than 7 on the HDRS was used as cut-off to define the presence of depressive symptoms [33]. In order to have a common measure for depressive symptoms, given that almost all patients were administered the HDRS, BDI scores were converted into HDRS scores according to current equating norms [37] for homogeneity purposes.

Patients with ALS were therefore classified as: patients who had neither apathy nor depressive symptoms (ALSn), patients who presented apathy in absence of depressive symptoms (ALSa), patients with depressive symptoms without apathy (ALSd), and patients with both apathy and depressive symptoms (ALSad).

### Statistical analysis of sociodemographic, clinical, and cognitive data

Sociodemographic and clinical data were compared among groups using ANOVA models. Pearson’s Chi-square test was used for the analysis of categorical variables. Differences in the performances at the standard neuropsychological evaluation were assessed using ANCOVA models accounting for age, sex and education, followed by post hoc pairwise comparisons (Bonferroni-corrected for multiple comparisons, p < 0.05). Differences in terms of presence of apathy and depressive symptoms in ALS patients with limb and bulbar onset were assessed with using Student’s t-test.

### MRI acquisition

MRI scans were obtained at the IRCCS Scientific Institute San Raffaele, Milan, Italy, using two different 3.0 T Philips scanners (Ingenia CX; Intera). Using a 3.0 T scanner (Ingenia CX, Philips), the following brain MRI sequences were obtained for 29 patients and 28 healthy controls: 3D T1-weighted (TFE) (TR = 7 ms; TE = 3.2 ms; flip angle = 9 [degrees]; 204 contiguous sagittal slices with voxel size = 1 × 1 × 1 mm, matrix size = 256 × 240, FOV = 256 × 240 mm^2^); 3D FLAIR (TR = 4800 ms; TE = 267 ms; TI = 1650 ms; ETL = 167; NEX = 2; 192 contiguous sagittal slices with voxel size = 0.89 × 0.89 × 1 mm, matrix size = 256 × 256, FOV = 256 × 256 mm^2^); 3D T2-weighted (TR = 2500 ms; TE = 330 ms; ETL = 117; NEX = 1; 192 contiguous sagittal slices with voxel size = 0.89 × 0.89 × 1 mm, matrix size = 256 × 258, FOV = 256 × 256 mm^2^); and T2* weighted (GE-EPI) sequence for resting-state functional MRI (RS-fMRI) (TR = 1567 ms; TE = 35 ms; flip angle = 70; MB = 2; SENSE = 2; FOV = 240 × 240; pixel size = 2.5 × 2.5 mm; slice thickness = 3 mm; 320 sets of 48 contiguous axial slices; acquisition time = 8’ and 32’’).

Using a 3.0 T scanner (Intera, Philips), the following brain MRI sequences were obtained for 53 patients and 18 healthy controls: T2-weighted spin echo (repetition time [TR] = 3500 ms; echo time [TE] = 85 ms; echo train length = 15; flip angle = 90 [degrees]; 22 contiguous, 5-mm-thick, axial slices; matrix size = 512 × 512; field of view [FOV] = 230 × 184 mm^2^); fluid-attenuated inversion recovery (TR = 11 s; TE = 120 ms; flip angle = 90 [degrees]; 22 contiguous, 5-mm-thick, axial slices; matrix size = 512 × 512; FOV = 230 mm^2^); 3D T1-weighted fast field echo (FFE) (TR = 25 ms; TE = 4.6 ms; flip angle = 30 [degrees]; 220 contiguous axial slices with voxel size = 0.89 × 0.89 × 0.8 mm, matrix size = 256 × 256, FOV = 230 × 182 mm^2^); and T2*-weighted single-shot echo planar imaging sequence for RS-fMRI (TR = 3000 ms; TE = 35 ms; flip angle = 90°; FOV = 240 × 240 mm^2^; matrix size = 128 × 128; slice thickness = 4 mm; 200 sets of 30 contiguous axial slices; acquisition time = 10 min).

Before starting the RS-fMRI scanning, the technician talked with the participants through their earphones instructing them to remain motionless, to keep their eyes closed, not to fall asleep, and not to think about anything in particular. At the end of the RS-fMRI acquisition, the technician talked again with the participants asking whether they remained awake during the sequence.

### MRI analysis

MRI data were analyzed at the Neuroimaging Research Unit, IRCCS San Raffaele Scientific Institute, Milan, Italy. An experienced observer, blinded to participants’ identity and diagnosis, performed MRI analysis. Data were acquired on two different 3 T Philips scanners. Since the number of patients scanned on each system was small, we combined the two datasets to increase the overall statistical power. In all analyses (ANOVAs and regression models), scanner was included as a covariate to control for any systematic signal differences between the two instruments.

*Brain parcellation.* Each brain image was parcellated into 220 similarly sized regions, as previously described [38]. We adopted a 220‐node gray-matter parcellation to balance high spatial resolution with anatomical interpretability. Starting from the 116‐region AAL atlas, we applied a constrained K-means algorithm (maximum *N* = 250) to split each AAL region of interest (ROI) into similarly sized, spatially contiguous parcels, selecting the solution (262 ROIs) with the lowest interquartile-to-median volume ratio. We then excluded 42 cerebellar parcels to yield 220 cortical gray matter (GM) regions. This approach ensures uniform node volumes while respecting established anatomical landmarks and has been employed in several prior connectomic studies [38–40]. The coregistrations between each subject’s T1-weighted image and MNI152 standard space (linear and non-linear using FMRIB’s Linear Image Registration, FLIRT, and FMRIB’s Non-linear Image Registration, FNIRT Tools, respectively, as implemented in FMRIB Software Library, FSL [FSLv5.0.9; http://www.fmrib.ox.ac.uk/fsl]) and between subject’s RS-fMRI and T1-weighted images (linear coregistration as implemented in FLIRT) were calculated and concatenated in order to move the 220 GM ROIs into the subject’s RS-fMRI space.

*RS-fMRI preprocessing.* RS-fMRI data were preprocessed using FSL and the Statistical Parametric Mapping software package (SPM12; http://www.fil.ion.ucl.ac.uk/spm/) running on Matlab 7.6. The first part of the preprocessing was performed using FSL and included: (1) removal of the first four volumes to allow for signal equilibration; (2) head movement correction by volume-realignment to the middle volume using Motion Correction FLIRT (MCFLIRT); and (3) removal of non-brain tissue. Linear detrending and band-pass filtering between 0.01 and 0.08 Hz were performed using REST software (http://resting-fmri.sourceforge.net/) to partially remove low-frequency drifts and physiological high-frequency noise. Using REST, non-neuronal sources of synchrony between RS-fMRI time series and motion-related artifacts were minimized by regressing out the six motion parameters estimated by MCFLIRT, and the average signals of the ventricular cerebrospinal fluid and white matter.

*Functional connectome reconstruction.* Functional connectivity matrices were obtained on the basis of correlation analysis. Mean time series were extracted from each ROI by averaging the signal from all voxels within each region. RS-fMRI data were masked with the subject’s GM map in order to consider only voxels corresponding to GM and avoid the effect of atrophy. Cortical GM was segmented using SPM12, while basal ganglia (i.e., bilateral caudate, globus pallidus, putamen, and thalamus), hippocampus, and amygdala maps were obtained using FIRST in FSL. The Pearson correlation coefficient between the mean time series of each node pair, indicating the level of functional connectivity between regions i and j, was entered into cell c(i,j) of the matrix. Pearson correlation coefficients were then converted to z-scores using the Fisher r-to-z transformation. Negative values were set as “NaN” to mark these brain regions as unconnected. This choice was made due to the limited interpretability and potential instability of negative functional connections in resting-state data, as well as to maintain methodological consistency with prior literature [41]. Functional connections of each subject were required to be present in a structural connectivity matrix of an independent healthy control sample (N = 90, mean age 62.3 ± 8.07 years, 51 women/39 men), that is, we measured functional interactions only where an anatomical connection between two areas occurs in the independent healthy control sample, as previously reported [38].

*Global brain and lobar network measure analysis.* Global and mean lobar functional network characteristics were explored using the Brain Connectivity MATLAB toolbox (brain-connectivity-toolbox.net). Network metrics, including nodal strength, characteristic path length, local efficiency, and clustering coefficient, were assessed to characterize the topologic organization of global brain and lobar networks in ALS patients and healthy controls [42, 43]. In order to investigate the network characteristics in different areas of the brain, the 220 regions of interest from both hemispheres were grouped into six anatomical macro-areas (hereafter referred to as brain lobes): temporal, parietal, occipital, frontoinsular, basal ganglia, and sensorimotor areas [38]. Global and local metrics were compared between all ALS patients and healthy controls using ANOVA models, followed by post hoc pairwise comparisons, Bonferroni-corrected for multiple comparisons (*P* < 0.05, SPSS Statistics 24.0). Comparisons were adjusted for MRI scanner.

*Regional functional connectivity analysis.* Network-based statistics (NBS) [38, 44] were performed to assess regional functional connectivity alterations in all ALS patients and matched healthy controls at a significance level of *P* < 0.05. For each between-group comparison, the test statistic is computed for each functional connection, obtaining the corresponding P values. Each connection that shows a *P* value < 0.05 is considered to be part of the functional altered pattern. At this point, since the inherent massive number of multiple comparisons involved and the great effort in testing the normality of each connection, a permutation test is used to ascribe a *P* value controlled for the family-wise error (FWE) to the functional altered pattern considered. The permutation test re-samples N times the total number of observations in a population sample to build an empirical estimate of the null distribution from which the test statistic has been drawn. A corrected *P* value was calculated using MRI scanner-adjusted permutation analysis (5.000 permutations).

*Correlation analysis.* In order to identify brain network alterations associated to apathy and depressive symptoms in ALS, in all ALS patients the relationship of global and lobar network metrics with HDRS and apathy composite score was tested using the Pearson correlation analysis (*p* < 0.05, corrected for multiple comparisons). HDRS and apathy composite score were also correlated with regional functional connectivity data by means of Pearson correlations. The analyses were adjusted for ALSFRS-R and MRI scanner.

*Patient group comparisons.* To further investigate the findings of distinct network alterations associated with apathy and depression resulting from the correlation analysis, global brain and lobar network measures analysis and NBS were performed to compare healthy controls and each ALS group defined on the presence of apathy and/or depressive symptoms (ALSn, ALSa, ALSd). Due to the very small sample size of the ALSad group, this was excluded from the between-group comparisons to avoid yielding unreliable results. On the basis of the results of the comparisons between healthy controls and each ALS group, and to increase the statistical power, ALS patients were grouped in those with (ALSa + ALSad, *N* = 20) and without (ALSn + ALSd, *N* = 62) apathy. Global brain and lobar network measures analysis and NBS were repeated to compare ALS patients with and without apathy and healthy controls. All comparisons between ALS subgroups were adjusted for ALSFRS-R and MRI scanner. Analyses were run via IBM® SPSS® Statistics 24 [SPSS Inc., Chicago, IL]. The significance threshold was set at α = 0.05.

## Results

### Demographic, clinical and neuropsychological features

Sociodemographic and clinical characteristics of healthy controls and ALS patients, stratified according to the presence of apathy and depression, are summarized in Table 1. Out of the 82 patients, 42 patients were ALSn, 14 ALSa, 20 ALSd and 6 ALSad. Patient groups and healthy controls were matched for age, sex and education and showed similar global cognitive functioning (MMSE). ALSad patients had a greater disease severity, when compared to ALSn and ALSa, and higher disease progression rate compared to ALSn, ALSa and ALSd. Compared with ALSa and ALSad, ALSd patients did not carry genetic mutations. Limb (*N* = 59) and bulbar onset (*N* = 21) patients did not show differences in terms of apathy and depressive symptoms (*p* > 0.05). Patients with mixed onset (*N* = 2), namely those who present with both bulbar and limb symptoms either at onset or shortly after, were not included in this analysis, as this phenotype might reflect a more diffuse or aggressive disease presentation at baseline. Regarding handedness, among the three non-right-handed patients, the two left-handed individuals were classified as ALSn and ALSd, respectively, while the ambidextrous patient was classified as ALSa.

**Table 1:** Sociodemographic and clinical features

	HC	All ALS	ALSn	ALSa	ALSd	ALSad
N	46	82	42	14	20	6
Sex [women]	27 (59%)	41 (50%)	20 (48%)	7 (50%)	11 (55%)	3 (50%)
Age at MRI [years]	62.42 ± 5.68	60.22 ± 10.95	60.79 ± 9.97	60. 21 ± 13.55	59.29 ± 11.17	59.42 ± 13.01
Education [years]	11.41 ± 4.20	11.23 ± 4.01	11.38 ± 4.27	10.14 ± 3.90	11.65 ± 3.77	11.33 ± 3.67
Disease duration [months]	–	21.59 ± 21.93	22.33 ± 26.10	17.29 ± 11.54	25.43 ± 20.82	13.67 ± 4.18
Site of onset [bulb/limb/limb + bulb]	–	21/59/2	11/30/1	4/10/0	2/17/1	4/2/0
ALSFRS-R, 0–48	–	38.82 ± 6.16	40.19 ± 5.08^**§**^	40.14 ± 5.48^**§**^	36.93 ± 5.16	31.83 ± 11.41
Disease progression rate	–	0.65 ± 0.63	0.60 ± 0.57^**§**^	0.61 ± 0.43^**§**^	0.58 ± 0.36^**§**^	1.45 ± 1.44
Type of scanner [scan 1/scan 2]	18/28	53/29^**#**^	31/11^**#§**^	11/3^**#§**^	10/10	1/5
Gene mutations	–	16 (19.5%)^**°**^ (9 C9orf72 1 C9orf72 + TDP-43 2 SOD1 4 TDP-43)	9 (21.4%)^**°**^ (6 C9orf72 1 SOD1 2 TDP-43)	6 (42.8%)^**°**^ (3 C9orf72 1 SOD1 1 TDP-43 1 TDP-43 + C9orf72)	0 (0%)	1 (16.6%)^**°**^ (1 TDP-43)
Presence of cognitive impairment	–	21 Bi (25%) 12 Ci/CiBi (14%)	6 Bi (14%) *^§^ 5 Ci (11%)	10 Bi (71%) 4 CiBi (28%)	2 CiBi (10%)*^§^	5 Bi (83%) 1 CiBi (16.6%)
MMSE	29.79 ± 1.37	29.67 ± 1.53	28.71 ± 1.37	28.93 ± 1.07	28.55 ± 1.82	28.17 ± 2.56
Digit span, forward	5.91 ± 10.11	5.67 ± 1.13	5.63 ± 1.13	4.82 ± 0.98^**#**^	6.21 ± 1.13*	5.83 ± 0.41
RAVLT, immediate recall	47.21 ± 7.53	44.11 ± 11.59	44.03 ± 11.40	38.18 ± 9.44	48.79 ± 11.96	40.00 ± 11.25
RAVLT, delayed recall	9.78 ± 2.44	9.29 ± 3.17	9.78 ± 3.13	8.00 ± 2.68	9.63 ± 3.20	7.20 ± 3.70
Card Sorting Test, perseverations [percentages]	0.07 ± 0.02	0.12 ± 0.01^**#**^	0.13 ± 0.13	0.15 ± 0.13	0.09 ± 0.10	0.07 ± 0.03
Phonemic fluency	36.54 ± 10.65	33.31 ± 10.83	31.57 ± 9.77	34.42 ± 10.60	37.32 ± 11.34	29.60 ± 16.32
Semantic fluency	45.81 ± 11.18	41.32 ± 9.31^**#**^	41.56 ± 8.14	40.86 ± 10.44	42.84 ± 8.46	35.00 ± 7.03
Digit span, backward	4.38 ± 1.40	4.13 ± 1.31^**#**^	4.24 ± 1.30	3.36 ± 1.20^**#**^	4.50 ± 0.92	3.80 ± 2.28
Cognitive estimation task	–	14.28 ± 4.22	14.24 ± 3.82	14.45 ± 4.30	13.84 ± 4.37	15.80 ± 6.69
BADA, nouns	–	29.67 ± 0.82	28.97 ± 1.38	29.00 ± 1.15	29.00 ± 0.89	29.00 ± 0.81
BADA, verbs	–	27.33 ± 0.81	26.84 ± 1.92	26.2 ± 1.75	27.27 ± 1.42	26.5 ± 1.29
FBI	–	3.11 ± 4.10	2.59 ± 4.05	5.25 ± 5.55	1.74 ± 2.28^**§**^	7.00 ± 2.00
FBI, item 1, apathy	–	0.40 ± 0.60	0.27 ± 0.45*^§^	0.82 ± 0.75	0.21 ± 0.42*^§^	1.20 ± 0.84
FBI, item 2, aspontaneity	–	0.28 ± 0.59	0.14 ± 0.35^**§**^	0.55 ± 0.93	0.21 ± 0.42^**§**^	1.00 ± 1.00
ALSFTD-Q	–	11.20 ± 11.10	7.26 ± 7.50*^§^	19.15 ± 14.78	8.64 ± 7.53	20.33 ± 12.82
ALSFTD-Q, item 1, loss of interest	–	0.53 ± 1.05	0.16 ± 0.37*^§^	1.46 ± 1.66	0.14 ± 0.36*	1.33 ± 1.37
ALSFTD-Q, item 7, social withdrawal	–	0.91 ± 1.31	0.23 ± 0.42*^§^	2.38 ± 1.39	0.29 ± 0.46*^§^	2.67 ± 1.37
HDRS	–	5.83 ± 4.88	3.00 ± 2.22^**§°**^	3.55 ± 1.97	11.50 ± 4.90*^§^	10.40 ± 1.67
BDI	4.30 ± 4.07	7.63 ± 4.07^**#**^	4.43 ± 3.36	5.33 ± 1.53	12.50 ± 4.72	–
Apathy rating scale	5.32 ± 3.89	–	–	–	–	–

Values reflect means ± standard deviations or frequencies. P values (*p* < 0.05) refer to ANOVA models followed by post hoc pairwise comparisons (Bonferroni-corrected for multiple comparisons, considering a family of six comparisons when comparing the four ALS groups, and a family of ten comparisons when comparing the five groups). Analyses on neuropsychological variables were performed accounting for age, sex and education. ^**#**^ = compared to HC; * = compared to ALSa; ^**§**^ = compared to ALSad; ^**°**^ = compared to ALSd. *ALS* Amyotrophic Lateral Sclerosis, ALSa patients who presented apathy in absence of depressive symptoms, *ALSad* patients with both apathy and depressive symptoms, *ALSd* patients with depressive symptoms without apathy, *ALSn* patients who had neither apathy nor depressive symptoms, *ALSFTD-Q* ALS-Frontotemporal lobar degeneration Questionnaire, *BADA* battery for the analysis of aphasic deficits, *BDI* Beck Depression Inventory, *FBI* Frontal Behavioural Inventory, *HC* Healthy Controls; *HDRS* Hamilton Depression Rating Scale, *MMSE* Mini-Mental State Examination, *RAVLT* Rey Auditory Verbal Learning Test. Only 16 patients completed the BDI: 7 ALSn, 3 ALSa, and 6 ALSd. For this reason, for BDI no between-group comparisons were performed

Concerning the neuropsychological features, healthy controls and ALS patients performed similarly in almost all considered tests, except for a worse performance of ALS patients in the digit span backward, perseverations at the Card Sorting Test and in the semantic fluency test (Table 1). Furthermore, ALSa patients showed worse scores at the forward and backward digit span compared to healthy controls and worse scores at the forward digit span compared to ALSd. As expected, patients with apathy and depression showed differences in terms of behavioural and depressive symptoms (Table 1). When classified according to Strong et al. criteria [5], patients with apathy demonstrated a higher percentage of cognitive impairment compared to those without apathy (Table 1). Of the 26 patients with ALS and depressive symptoms (ALSd and ALSad), only three were on antidepressant treatment at the time of MRI (triazolam 0.125 mg die, and escitalopram 20 mg die, sertraline 50 mg die, respectively). In general, patients included in the present study underwent MRI and neuropsychological assessment during hospitalization, after which an antidepressant was prescribed if needed.

### MRI findings

*Between-group comparisons.* The comparison between all ALS patients and healthy controls did not show differences in functional graph properties at global and lobar level, nor in the regional connectivity (NBS).

**Fig. 1:**
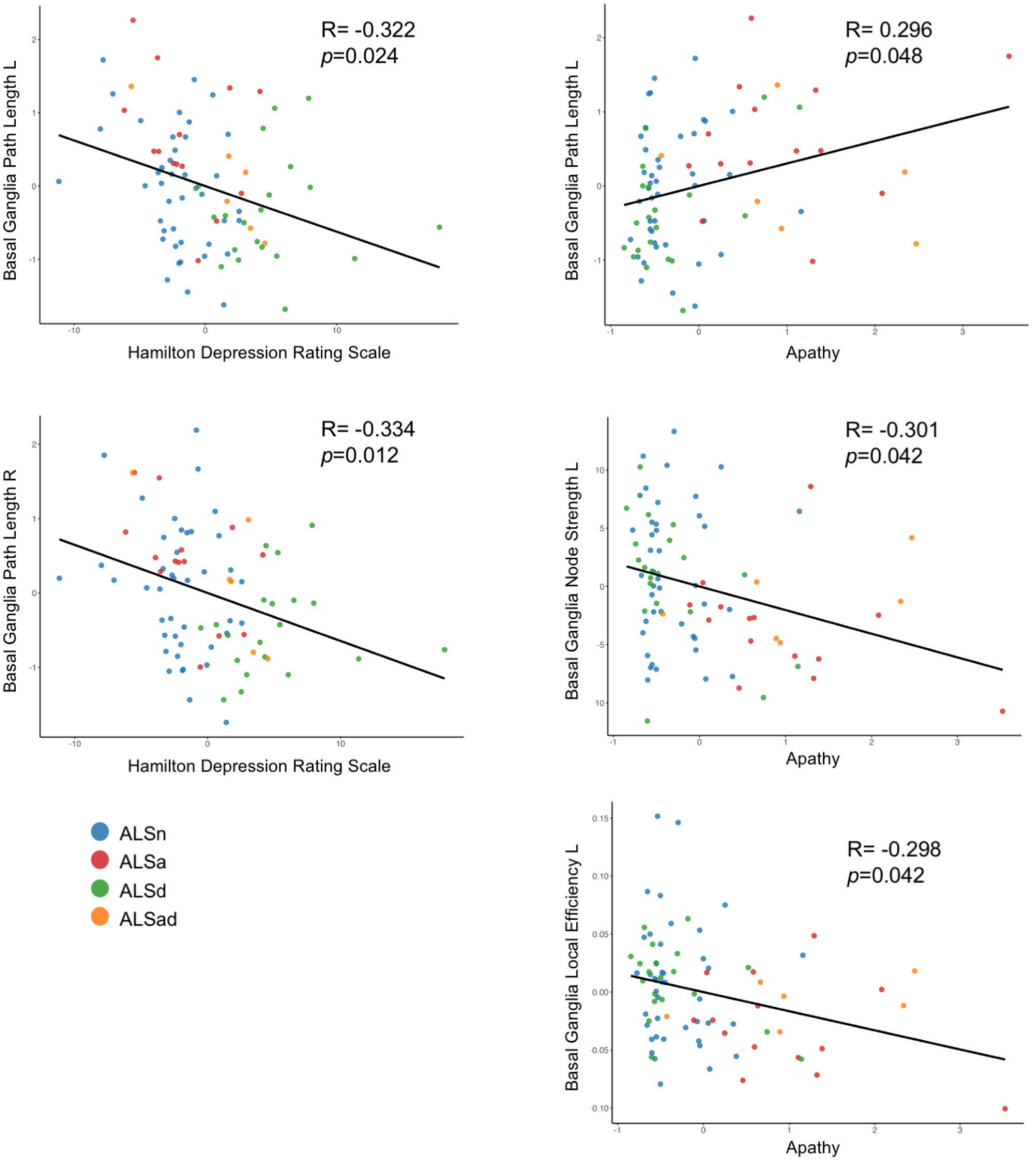
Relationship of lobar network metrics with Hamilton Depression Rating Scale and apathy composite score. The relationship was tested in all patients, using the Pearson correlation analysis (*p* < 0.05, corrected for multiple comparisons, considering a family of six comparisons, corresponding to the number of the brain lobes). The analysis was adjusted for ALSFRS-R and MRI scanner. *ALS* Amyotrophic Lateral Sclerosis, *ALSa* patients who presented apathy in absence of depressive symptoms, *ALSad* patients with both apathy and depressive symptoms, *ALSd* patients with depressive symptoms without apathy, *ALSn* patients who had neither apathy nor depressive symptoms, *L* left, *R* right

*Correlation analysis.* Correlation analyses showed that in all patients with ALS, increased depressive symptoms (defined by the HDRS scores) were related with a reduced lobar path length within the bilateral basal ganglia network. Increased apathy (defined through the composite apathy score) was related with increased lobar path length, and decreased nodal strength and local efficiency within the left basal ganglia network (Fig. 1). The present findings were not influenced by the presence of ALS patients with genetic mutations, whose distribution was similar to that of sporadic cases (see Supplementary Fig. 1). Correlation analysis between HDRS and apathy composite score with regional functional connectivity (NBS) yielded no significant results.

**Fig. 2:**
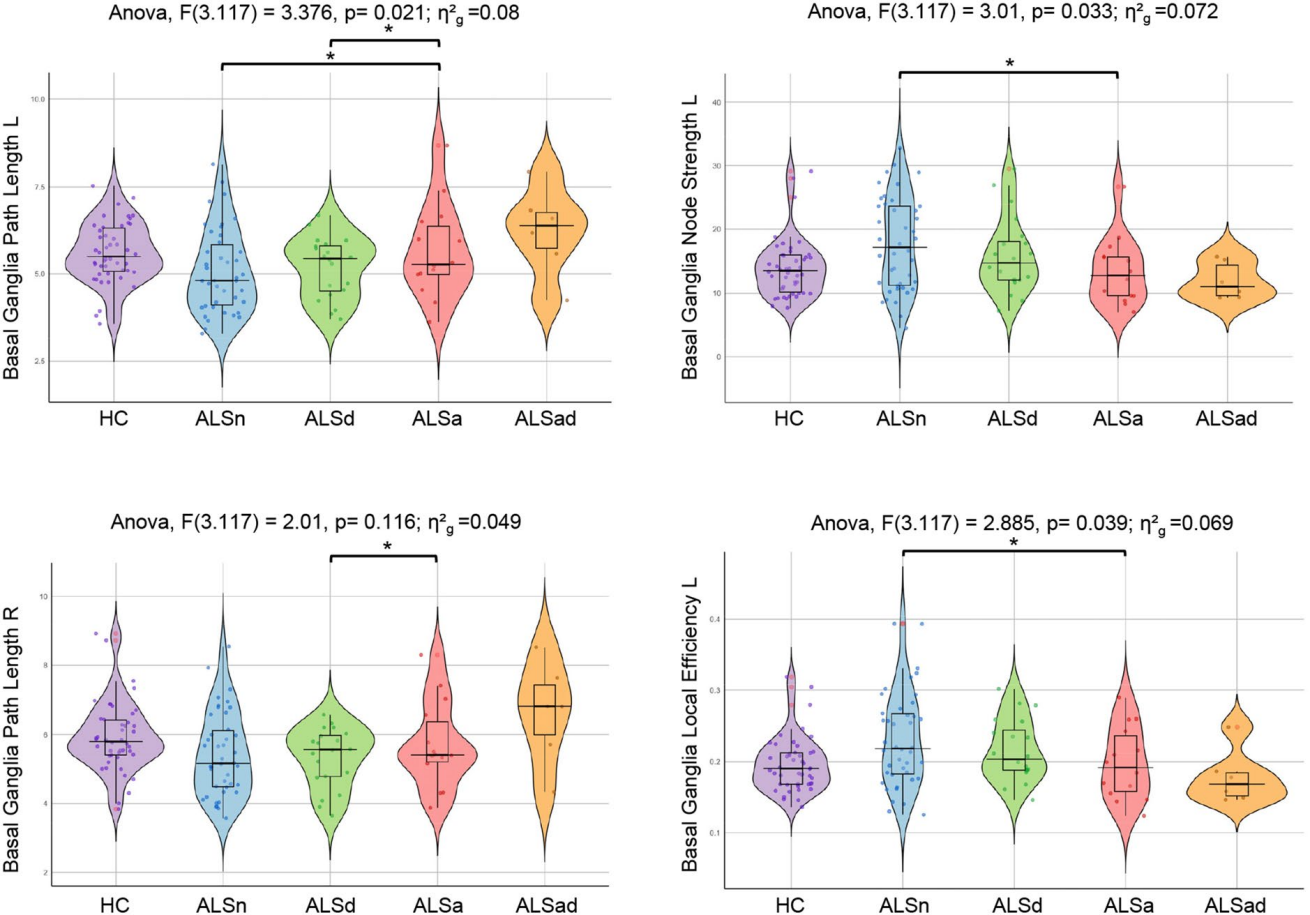
Functional connectome differences between ALS patients groups and healthy controls. The horizontal line in each box plot of the violin plots represents the median, the 2 lines just above and below the median represent the 25th and 75th percentiles. *p < 0.05, Bonferroni-corrected for multiple comparisons (considering a family of six comparisons when comparing all four groups, and a family of three comparisons when comparing the patient groups only). Due to the very small sample size, ALSad group was excluded from the between-group comparisons to avoid yielding unreliable results. Comparisons between patient groups were adjusted for ALSFRS-R and MRI scanner. Comparisons between patients and HC were adjusted for MRI scanner. *ALS* Amyotrophic Lateral Sclerosis, *ALSa* ALS patients who presented apathy in absence of depressive symptoms, *ALSad* ALS patients with both apathy and depressive symptoms, *ALSd* ALS patients with depressive symptoms without apathy, *ALSn* ALS patients who had neither apathy nor depressive symptoms, *HC* healthy controls; *L* left, *R* right

**Fig. 3:**
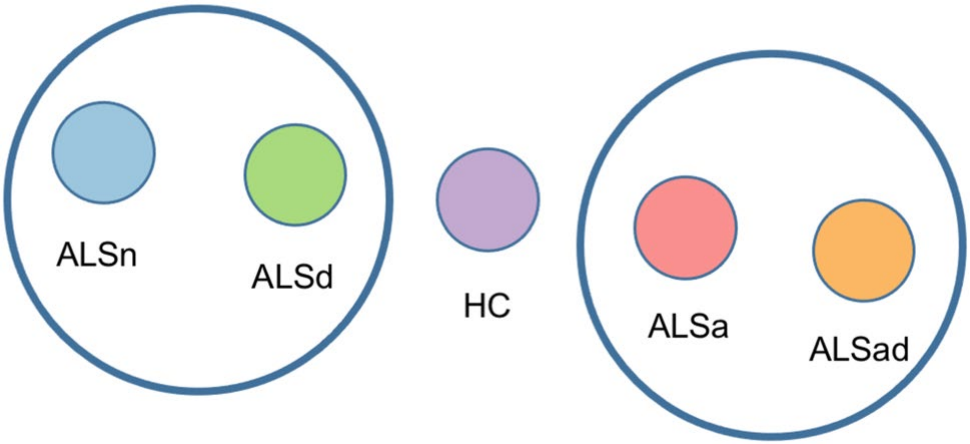
Brain network connectivity patterns resulting from between-group comparisons. High functional connectivity was observed in the ALSn and ALSd groups, reduced functional connectivity in ALSa and ALSad patients, HC showed intermediate functional connectivity levels. *ALSa* ALS patients who presented apathy in absence of depressive symptoms, *ALSad* ALS patients with both apathy and depressive symptoms, *ALSd* ALS patients with depressive symptoms without apathy, *ALSn* ALS patients who had neither apathy nor depressive symptoms, *HC* healthy controls

*Patient group comparisons.* Comparing healthy controls and each group of ALS patients defined on the presence of apathy and/or depressive symptoms (ALSn, ALSa, ALSd), no significant differences emerged in functional graph properties at global level. By contrast, at lobar level, ALSa cases had an increased path length and reduced local efficiency and nodal strength in the left basal ganglia network compared to ALSn, and an increased path length in the bilateral basal ganglia network compared to ALSd (Fig. 2). In Fig. 2, for completeness, we also represented the functional connectivity of ALSad group. These findings showed a pattern of brain network metrics, with higher functional connectivity in ALSn and ALSd patients, intermediate values in healthy controls, and ALSa and ALSad patients showing reduced functional connectivity (Fig. 3).

**Fig. 4:**
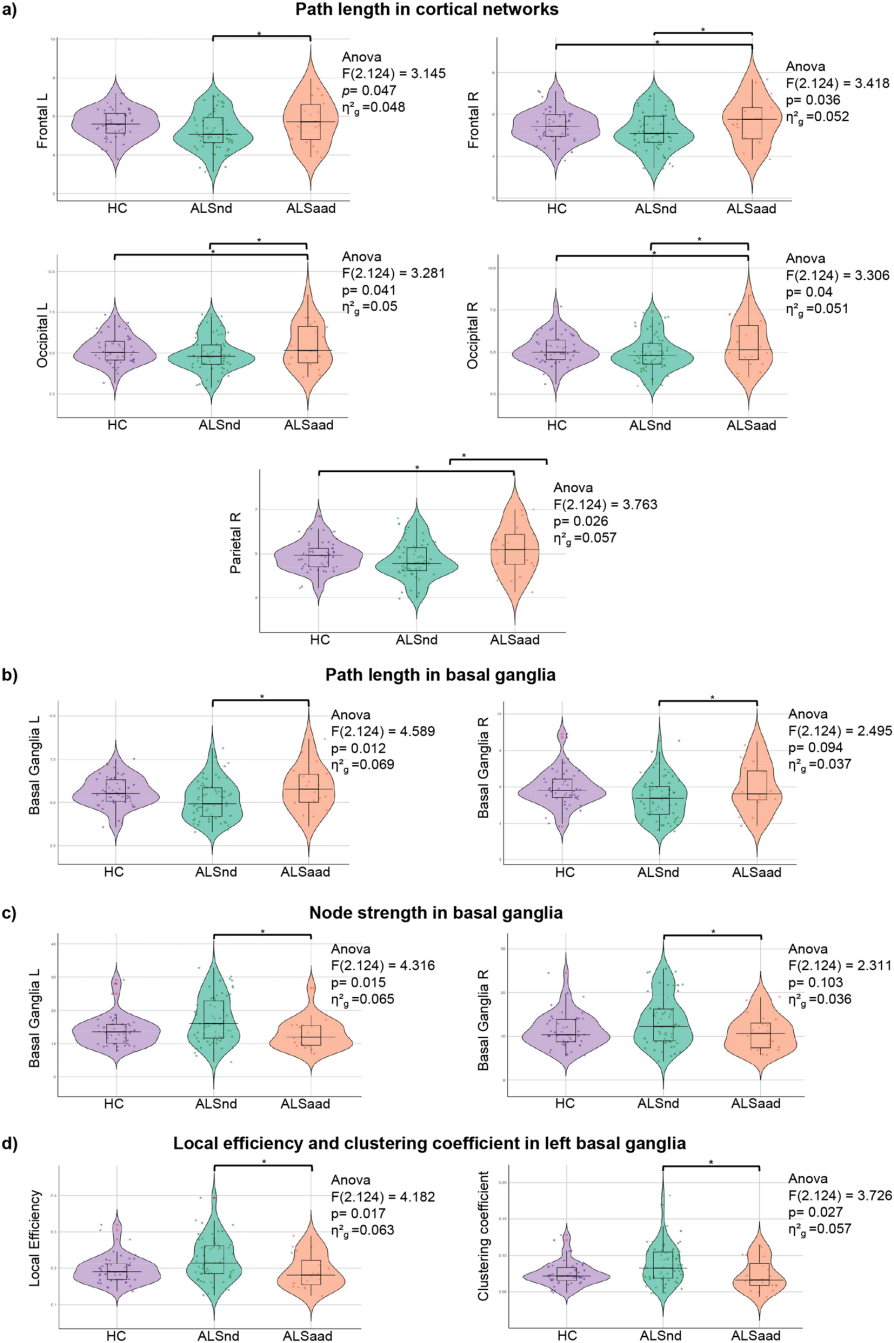
Functional connectome differences in cortical networks (**a**) and basal ganglia (**b, c, d**) between all apathetic (ALSa + ALSad), all non-apathetic patients (ALSn + ALSd) and healthy controls. The horizontal line in each box plot of the violin plots represents the median, the 2 lines just above and below the median represent the 25th and 75th percentiles. *p < 0.05, Bonferroni-corrected for multiple comparisons (considering a family of three comparisons when comparing the three groups; no correction was applied when comparing the two patient groups). Comparisons between patient groups were adjusted for ALSFRS-R and MRI scanner. Comparisons between patients and HC were adjusted for MRI scanner. *ALS* Amyotrophic Lateral Sclerosis, *ALSaad* apathetic patients, *ALSnd* non-apathetic patients, *HC* healthy controls, *L* left; *R* right

Comparisons between ALS patients with apathy (ALSa + ALSad), patients without apathy (ALSn + ALSd) and healthy controls showed that, at global level, patients with apathy exhibited a significant increased path length compared to patients without apathy (p = 0.04). At lobar level, patients with apathy, compared to healthy controls and patients without apathy, exhibited increased path length in right parietal, right frontal, and bilateral occipital networks (Fig. 4). Moreover, compared to non-apathetic patients, apathetic cases showed increased path length in the left frontal network and in the basal ganglia network bilaterally. Finally, relative to non-apathetic patients, they further showed reduced nodal strength in the basal ganglia network bilaterally, and decreased local efficiency and clustering coefficient in the left basal ganglia network. The present findings were not influenced by the presence of ALS patients with genetic mutations, whose distribution of functional nodal properties was similar to that of sporadic cases (see Supplementary Fig. 2).

## Discussion

This study aimed to disentangle the functional neural correlates of apathy and depression in ALS patients through a connectome-based approach. Our findings provide evidence that apathy and depression in ALS are both associated with brain network alterations, specifically in the basal ganglia, however with differently altered network properties, reinforcing the notion that these two neuropsychiatric symptoms have partially overlapping but independent neural substrates.

Graph theoretical measures were chosen over simple pairwise connectivity because they capture higher order properties of network organization, which allow the identification of subtle, spatially distributed alterations in network structure that may underlie specific neuropsychiatric symptoms. In particular, apathy and depression are thought to reflect disruptions in different aspects of large-scale brain organization, which graph metrics can better capture than individual connections alone.

ALS patient subgroups (ALSn, ALSa, ALSd, ALSad) were well-matched with healthy controls in terms of age, sex, and education. Global cognitive functioning was comparable across all groups. However, when classified according to Strong *et* al. criteria [5], patients with apathy demonstrated a higher percentage of cognitive impairment compared to those without apathy. More specifically, ALSa patients performed significantly worse than healthy controls on both the forward and backward digit span tasks, and significantly worse than ALSd patients on the forward digit span. The digit span tests are measures of attention and working memory, and these align with studies showing that ALS patients with apathy have executive function deficits [3]. These findings further support the notion that apathy, particularly in ALS, may be linked to frontal-subcortical dysfunction, which affects cognitive processes such as initiation and sustained attention.

Apathy in ALS was also linked to increased path length, reduced nodal strength, and decreased local efficiency in the basal ganglia network. Among the three subtypes of apathy, namely emotional-affective, cognitive and initiation, the one more associated with basal ganglia alterations is the initiation apathy, in which self-generated actions are significantly diminished, while responses to external stimuli remain relatively intact [45]. Interestingly, initiation apathy is the most common apathy subtype observed in ALS patients [2, 46]. Thus, the alterations we identified in the basal ganglia network may correspond to difficulties in initiating and maintaining cognitive and motor activities [13]. Future studies adopting the apathy subtype framework are required to better understand the specific mechanisms underpinning basal ganglia dysfunction in ALS and how initiation apathy relates to these connectivity alterations. Our analysis also revealed that, compared to non-apathetic patients and controls, patients with apathy exhibited altered functional connectivity in the parietal and occipital regions. Alterations in the basal ganglia, frontal, and parietal regions align with previous findings of structural [9, 47] and fMRI studies [48] in ALS with apathy. These regions are involved in mediating motivation, goal-directed behavior and action planning [45]. The basal ganglia, in particular, interact with each other through circuits that are involved in motricity, but also coordination of cognition, behavioural and emotional functions [49].

ALS patients with depressive symptoms were characterized by a tendency towards an increased functional connectivity as compared to healthy controls and ALS with apathy. Furthermore, in all ALS cases, we showed that more severe depressive symptoms were related with a reduced path length within the bilateral basal ganglia network. These findings are consistent with studies reporting a significant increase in functional connectivity [50, 51] and a reduction in path length [52] across various regions, including the basal ganglia, in non ALS patients with a major depression condition compared to healthy controls. Our results are also in line with studies on neurodegenerative diseases, such as Parkinson’s disease, which found associations between altered connectivity in the basal ganglia and depressive symptoms [4]. In fact, basal ganglia are known to be involved in several higher order functions including also mood regulation, reward processing, and emotional reactivity [53]. The finding of reduced characteristic path length needs to be carefully interpreted. Indeed, in these patients, we observed reduced path length but not increased local efficiency, a measure that reflects the efficiency of a node in facilitating communication within its neighborhood. Reducing the path length in a network without improving local efficiency can enhance global connectivity, but not communication within neighborhoods of nodes, weakening local cohesion and reducing the network's ability to handle localized interactions effectively [52]. In the context of depression, this could mean faster communication between distant brain regions, but a compromised ability for nearby regions to coordinate and process information [54]. This imbalance may worsen symptoms such as emotional dysregulation and cognitive sluggishness [55].

Interestingly, we did not observe significant differences in the global network properties of ALS patients and control groups. This finding could be explained by two main factors. First, the absence of global, brain-wide impairments in connectivity for the ALS group suggests that alterations are localized [56]. In fact, our results show group differences mainly when considering specific anatomical macro-areas. Second, the pattern of brain network connectivity observed in the between-group comparisons of lobar network properties revealed a gradient, where ALSn and ALSd patients showed higher functional connectivity, ALSa and ALSad patients showed reduced connectivity, and healthy controls displayed intermediate values, positioned between the ALS subgroups. The lack of significant differences in global and lobar network properties (namely, within basal ganglia network) between healthy controls and ALS subgroups could therefore be explained by this intermediate placement of healthy controls, which may have reduced the statistical contrast with both extremes of the ALS group. Overall, this finding further supports the notion that network properties in ALS patients with and without apathy are differently altered.

The gradient observed in the pattern of brain network metrics among ALS groups aligns with findings from a recent connectome analysis in ALS. This study reported decreased functional connectivity in frontotemporal regions in ALS patients at risk of developing FTD, alongside increased functional connectivity in milder ALS cases [57]. The functional connectivity observed in our patients with depressive symptoms may resemble that of the milder cases, whereas the reduced functional connectivity in patients with apathy, or those with both apathy and depression, may align with patterns seen in ALS patients at risk of developing FTD. This underscores the importance of distinguishing between apathy and depression, as it may reveal critical differences in disease progression, further supporting the notion that apathy is indicative of a more aggressive disease phenotype with a poorer prognosis [6].

Despite the valuable insights gained from this study, there are several limitations that should be acknowledged. First, the sample size, particularly for the subgroups of ALS patients with both apathy and depression, was relatively small. Future studies should include larger cohorts and longitudinal designs. Second, we used two different approaches to assess depression and apathy symptomatology. Specifically, depressive symptoms were evaluated using a self-rated questionnaire, while apathy symptoms were identified based on caregiver-reported answers to semi-structured questionnaires. Of note, for the detection of apathy, we opted to use caregiver-rated scales, as they are particularly valuable in identifying apathy symptoms in patients who may have impaired awareness of their own lack of interest, emotional blunting and reduced initiative. Third, although we addressed the physiological confounds as described in the method section, we did not acquire concurrent cardiac and respiratory traces of the participants during fMRI scans. A further limitation is the use of two different MRI scanners. To control for potential systematic differences in signal characteristics between the two scanners, we included “scanner” as a covariate in all statistical models; however, the lack of advanced harmonization techniques may limit reproducibility.

In conclusion, this study provides evidence that apathy and depression in ALS are associated with distinct alterations in brain connectivity, particularly within the basal ganglia network. Apathy is characterized by disrupted connectivity and reduced network efficiency, while depression is linked to enhanced connectivity. These findings underscore the importance of distinguishing between apathy and depression in ALS and highlight the need for symptom-specific interventions. Further research is needed to explore the longitudinal trajectory of these symptoms and their impact on disease progression in ALS.

## Supplementary Information

Below is the link to the electronic supplementary material.

Supplementary file1 (PDF 103 KB)

## Data Availability

The anonymized dataset used and analyzed during the current study is available from the corresponding author upon reasonable request.
