# Supplementary material for: Brain functional connectivity changes in amyotrophic lateral sclerosis with apathy and depression
Source: J Neurol. 2025 Jul 14;272(8):509. doi: 10.1007/s00415-025-13247-1 (PMC12259760; doi:10.1007/s00415-025-13247-1)
Supplement: Supplementary file 1 — Supplementary file1 (PDF 103 KB) [file 415_2025_13247_MOESM1_ESM.pdf]

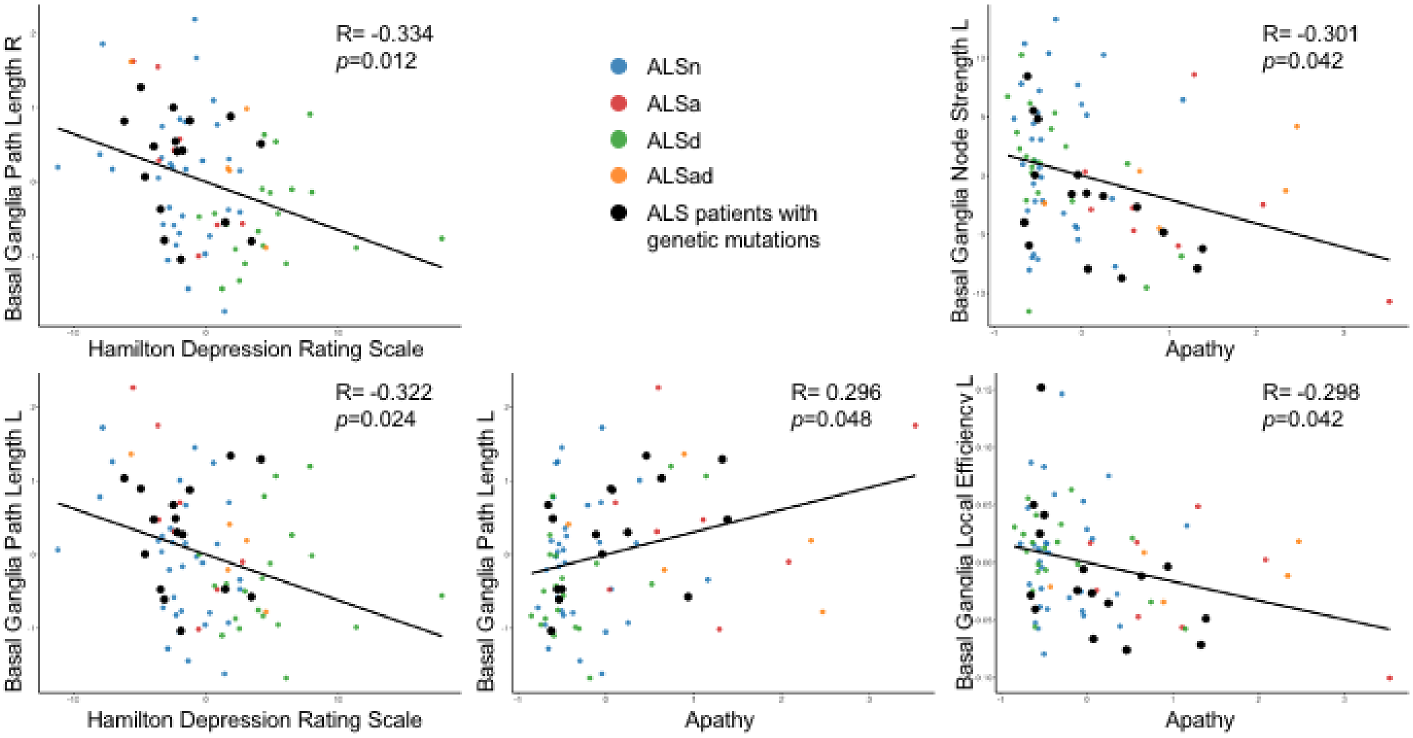

Supplementary Figure 1

Relationship of lobar network metrics with Hamilton Depression Rating Scale and apathy composite score, highlighting the distribution of ALS patients with genetic mutations (black, larger dots). The relationship was tested in all patients using the Pearson correlation analysis ($p < 0.05$, corrected for multiple comparisons). The analysis was adjusted for ALSFRS-R and MRI scanner. Abbreviations: ALS=Amyotrophic Lateral Sclerosis; ALSa=patients who presented apathy in absence of depressive symptoms; ALSad=patients with both apathy and depressive symptoms; ALSd=patients with depressive symptoms without apathy; ALSn=patients who had neither apathy nor depressive symptoms; L=left; R=right.

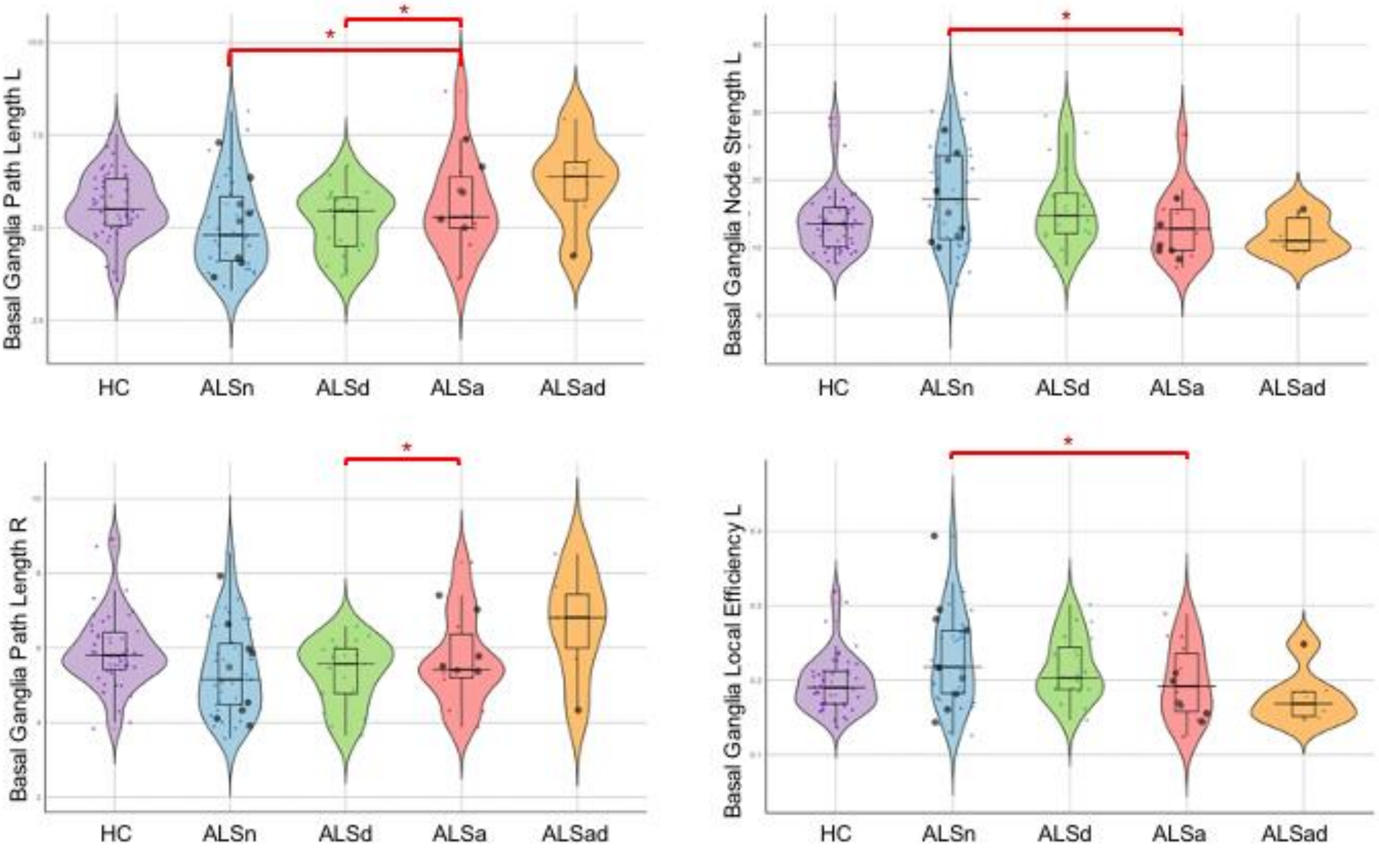

Supplementary Figure 2

Violin plots of the significant differences in functional graph properties at lobar level resulting from the comparisons between the four ALS patient groups and healthy controls, highlighting the distribution of functional nodal properties in ALS patients with genetic mutations (black, larger dots). The horizontal line in each box plot represents the median, the 2 lines just above and below the median represent the 25th and 75th percentiles. *$p < 0.05$, Bonferroni-corrected for multiple comparisons. The comparisons between patient groups were adjusted for ALSFRS-R and MRI scanner. The comparisons between patients and HC were adjusted for MRI scanner. Abbreviations: ALS=Amyotrophic Lateral Sclerosis; ALSa=ALS patients who presented apathy in absence of depressive symptoms; ALSad=ALS patients with both apathy and depressive symptoms; ALSd=ALS patients with depressive symptoms without apathy; ALSn=ALS patients who had neither apathy nor depressive symptoms; HC= healthy controls; L=left; R=right.
